# Combined inhaled corticosteroid and long-acting β2-adrenergic agonist therapy for noncystic fibrosis bronchiectasis with airflow limitation: An observational study: Notice of Retraction

**DOI:** 10.1097/MD.0000000000006039

**Published:** 2017-01-20

The publisher has requested that the article “Combined inhaled corticosteroid and long-acting β2-adrenergic agonist therapy for noncystic fibrosis bronchiectasis with airflow limitation: An observational study”^[1]^ be retracted. After publication, a reader brought to our attention inconsistencies in the methodology of the paper. The authors of the paper agreed that there were unforeseen inconsistencies in the data and the results were unreliable.
